# TCN-attention-HAR: human activity recognition based on attention mechanism time convolutional network

**DOI:** 10.1038/s41598-024-57912-3

**Published:** 2024-03-28

**Authors:** Xiong Wei, Zifan Wang

**Affiliations:** Wuhan Textile University, Wuhan, China

**Keywords:** Wearable sensor device, Human activity recognition, Time convolutional neural network, Attention mechanism, Knowledge distillation, Computer science, Information technology

## Abstract

Wearable sensors are widely used in medical applications and human–computer interaction because of their portability and powerful privacy. Human activity identification based on sensor data plays a vital role in these fields. Therefore, it is important to improve the recognition performance of different types of actions. Aiming at the problems of insufficient time-varying feature extraction and gradient explosion caused by too many network layers, a time convolution network recognition model with attention mechanism (TCN-Attention-HAR) was proposed. The model effectively recognizes and emphasizes the key feature information. The ability of extracting temporal features from TCN (temporal convolution network) is improved by using the appropriate size of the receiver domain. In addition, attention mechanisms are used to assign higher weights to important information, enabling models to learn and identify human activities more effectively. The performance of the Open Data Set (WISDM, PAMAP2 and USC-HAD) is improved by 1.13%, 1.83% and 0.51%, respectively, compared with other advanced models, these results clearly show that the network model presented in this paper has excellent recognition performance. In the knowledge distillation experiment, the parameters of student model are only about 0.1% of those of teacher model, and the accuracy of the model has been greatly improved, and in the WISDM data set, compared with the teacher's model, the accuracy is 0.14% higher.

## Introduction

In recent years, the widespread adoption of wearable sensor devices has led to a surge in research interest in intelligent sensing^1^. HAR (Human activity recognition)is a key area within intelligent sensing, primarily involving the extraction of activity features from sensor-generated time series data. Fundamentally, HAR involves feature extraction and intelligent analysis of sensor data^2^. HAR finds applications in diverse fields such as intelligent healthcare^3,4^, Smart Home^5–7^, monitoring systems^8^, human–computer interaction^9,10^and Fall detection^6^. Currently, HAR techniques can be broadly categorized into three types based on the data source: vision-based human behavior recognition, human activity recognition through environmental interaction, and sensor-based human activity recognition^11^. The sensors used for vision-based human behavior recognition are RGB camera^12–14^, and depth camera^15–17^, but they are too expensive to use Environmental interaction-based recognition is heavily influenced by environmental factors and has limited data availability. In terms of confidentiality and cost, wearable devices equipped with accelerometers, gyroscopes, magnetometers, and heart rate monitors have a significant advantage in human activity recognition due to their portability and ease of use^18^. Sensor-based human activity identification uses body sensors^19,20^, which is the focus of this paper.

However, effectively extracting relevant information from sensors and achieving high-precision and accurate human activity recognition has become a major research challenge^21^. Traditional methods for human activity recognition typically employ machine learning techniques^22^ such as K-nearest neighbor^23^, naive Bayes^24^, and random forest^25^. However, these traditional machine learning methods have notable limitations, particularly in the feature extraction stage, as they heavily rely on manual feature engineering and lack deep feature representation. In recent years, with the advent of deep learning^26^, the process of feature engineering has been greatly simplified. Deep learning methods, such as Convolutional Neural Networks (CNNs)^27^, Recurrent Neural Networks (RNNs)^28^, and Long Short-Term Memory (LSTM) networks^29^, have shown remarkable advancements in feature extraction and have gained widespread adoption in human activity recognition. The recognition of human activities typically involves four steps: sensor data collection, data preprocessing, data segmentation, feature extraction, and action classification. However, due to the diversity, complexity, and temporal nature of human movements, capturing the changes in human activities and selecting important features remain challenging tasks. To address these challenges, this paper proposes the following key contributions:

1. In the process of human activity recognition, the data read out by the sensor has a time rule, and when TCN is used for feature extraction, it is better at capturing temporal information, has a flexible receptive field, and uses attention to assign higher weights to important features, thus improving the effectiveness of the model.
2. We propose to use multi-scale TCN-attention-HAR to enhance the feature extraction capability of the model, and replace TCN with CNN network for comparison, which verifies that TCN plays a better role in the model.
3. Compared with traditional multi-channel CNN attention methods, experimental results evaluated on publicly available datasets WISDM, Pamap2, and USC-HAD show that the proposed model achieves performance improvements of 1.13%, 1.83% and 0.51%, respectively.
4. By using the method of knowledge distillation, the model presented in this article is used as a teacher model, which significantly improves the accuracy of the student model.

The structure of this paper is as follows: the first section describes the prospect and challenge of the proposal, the second section briefly reviews the work related to HAR, the third section mainly introduces the structure of the TCN-Attention-HAR model, the fourth section gives the experimental results and analysis, and the fifth section draws the conclusion.

## Related works

In recent years, image-based human activity recognition has been successfully deployed and applied. The widespread use of smart devices with embedded sensors brings new opportunities and challenges to the HAR field^30^. This section mainly describes the related research on sensing human activity recognition, which is mainly divided into machine learning and deep learning methods.

### Research on human body recognition

Jalal^31^ proposed a three-axis accelerometer human motion detection and recognition system based on multi-feature and random forest to evaluate the proposed model based on the HMP identification data set, and achieved a satisfactory recognition rate of 85.17%. Jalal^32^ Support Vector Machine 3D body postures for different RGB-D video sequences Jalal^33^ uses principal component analysis to process these features using hidden Markov model activity model recognition activities, with our method achieving 92.4% and 93.2% accuracy, respectively, in the case of public datasets. Kamal^34^ used improved hidden Markov Model (M-HMM) to identify different activities, and the recognition rate was 91.3%. Mahmood^35^ proposed the White Stag model, which achieved a weighted average recognition rate of 87.48% in UT-Interaction and 87.5% in BIT-Interaction, a weighted average recognition rate of 7.7% was achieved on the im-intensityinteractive 85 dataset. Using 3D-DCNN, Phyo^36^ was able to identify 95 percent of the 10 movements.

### Research on feature extraction

Jalal^37^ A mixture of four new features, namely, spatiotemporal features, energy-based features, shape-based angles and geometric features, and directional gradient motion orthogonal histograms, is presented Batool^38^ uses a biogeography optimization and re-weighted genetic algorithm to optimize and classify extracted features, which outperforms existing advanced methods compared with CMU-Multi-Modal Activity, WISDM and IMSB datasets, the recognition accuracy is 88%, 88.75% and 93.33% respectively. Jalal^39^ proposed the computation of multiple composite features, namely statistical features, Mel frequency cepstrum coefficients, and Gauss mixture model features, it achieves 1.88%, 25.93% and 95.96% accuracy on MOTIONSENSE, MHEALTH and the proposed self-annotated IM-AccGyro human–machine data sets, respectively. Jalal^40^ proposed encoding body shape information reflected in depth values into features, with an average recognition rate of 93.17% for 93 typical human activities Jalal^41^ extracted spatiotemporal multi-fusion features connecting three skeletal joint features and three body features, and trained the hidden Markov model by using code vector of multi-fusion features Adnan^42^ extracts distance location features and centroid distance features, and self-organized maps are used to identify different activities. Zin^43^ proposed a combination of spatiotemporal features with distance features, and the results of the experiment were tested in a random frame sequence in a dataset collected at an elderly care center.

### HAR research based on sensor data

In the past, the HAR field has generally been used for machine learning-based methods to detect human activity. Tharwat et al.^44^ used particle swarm optimization (PSO) algorithm to search for the optimal value of k parameter in KNN classifier, which improved the accuracy of KNN classifier. Fatima^45^ uses multiple support vector machine (SVM) cores to adopt a decision fusion mechanism to improve the accuracy of activity identification. Moriya et al.^46^ used locomotors integrated in various smart appliances to identify daily life, selecting a random forest model for activity classification with an accuracy of 68%. Bustoni et al.^47^ compared the performance of SVM, KNN and random forest machine learning methods, and the results showed that the SVM method with support vector classifier (SVC) and radial basis function (RBF) kernel could achieve the highest accuracy and recall rate. However, shallow machine learning methods use manual feature extraction, and the model relies on statistical features and distribution features, which greatly increases labor costs and affects the accuracy of activity classification.

In recent years, with the development of deep learning, traditional machine learning methods have been replaced by deep learning methods. Charissa et al.^48^ used this paper to propose a deep convolutional neural network (convnet). Using the inherent properties of active and one-dimensional time series signals, a method for extracting robust features automatically and data adaptively from raw data is provided. Marjan et al.^49^ proposed a new architecture based on 2D convolutional neural networks, which consists only of convolutional layers. By removing the pooling layer and adding steps to the convolutional layer, the computation time will be significantly reduced, while the model performance will not change. In some cases it was even improved, achieving an overall accuracy of 95.69% on the test set. Shao et al.^50^ proposed a real-time human activity classification method based on convolutional neural network (CNN), which uses CNN to carry out local feature extraction. Finally, the CNN, LSTM, BLSTM, MLP and SVM models were used for comparison on UCI and Pamap2 datasets. Li et al.^51^ designed a multi-channel CNN-GRU model, The model performance analysis was conducted on three benchmark datasets: WISDM, UCI-HAR, and PAMAP2, with accuracy rates of 96.41%, 96.67%, and 96.25%, respectively. Existing research work mainly uses traditional machine learning algorithms and deep learning algorithms to carry out. On the one hand, machine learning-related work relies too much on manual feature extraction, resulting in too tedious feature engineering stage. On the other hand, in the relevant work using deep learning, a part of the convolutional neural network is adopted, and the time-related feature extraction is not sufficient. Different from the above work, the TCN-Attention-HAR model proposed in this paper mainly uses the time convolutional neural network technology, which is better at capturing temporal dependencies, has a flexible receptive field, and uses the attention layer to fully extract the importance features of HAR.

### Research on classification and probability recognition

Zhang^52^ recommend deep neural networks (DNNs) for modeling the emission distribution of HMMs. Jalal^53^ recommend these features are processed by Principal component analysis for dimension reduction and k-mean clustering for code generation to make better activity representation The average recognition rate was up to 57.69% compared to using the IM-DailyDepthActivity data set. Jalal^54^ used probability-based incremental learning (PBIL) optimizer and K-Ary tree hash classifier to model different human activitiesThe experimental results show that our model outperformed existing state-of-the-art methods with accuracy rates of 94.23%, 94.07% and 96.40% over DALIAC, PAMPA2 and IM-LifeLog datasets, respectively. Jalal^55^ uses robust hybrid features and embedded hidden Markov model to identify video human activity Jalal^56^ using Linde–Buzo–Gray clustering algorithm to enhance the enhanced features and symbolic processing, in order to obtain better action recognition effect.

## Methods

### The overview of human activity recognition

The recognition process of human activities using a network model can be divided into four main steps: data acquisition, data processing, model training, and model evaluation. Data acquisition involves the use of sensors to collect acceleration signals, angular velocity signals, and gravity signals during human activities. Since sensor-based human activity recognition is a time series prediction classification problem, a sliding window method can be employed to segment the input signal data into signal windows. The window width and step size can be determined through experimentation.

**Figure 1:**
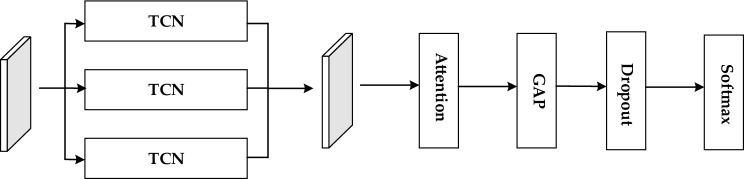
TCN-attention-HAR network structure.

The processed data is then input into the TCN-Attention-HAR model for training. As shown in Fig. 1, to extract more time-dependent information effectively, a time convolutional network is used to extract features from the preprocessed data at different scales. This enhances the model's recognition ability across various temporal aspects. The feature representation of each element in each channel is combined into a tensor, and feature fusion is performed across channels. This combined information is then passed through the Attention layer. Attention mechanism is used to strengthen the time correlation between one time node and other time nodes in TCN network model, and solve the problem that the TCN network model is too deep in layers and easy to neglect the important time sequence information, the model concentrates more on important and relevant features while suppressing irrelevant information. Subsequently, the locally relevant information is processed through the Global Average Pooling layer (GAP) to regularize the network structure and reduce the parameter input. Finally, the Softmax function is applied to estimate the categories of human activities.

During the human activity recognition process, the performance of the proposed TCN-Attention-HAR model is evaluated using accuracy, precision, recall rate, and F1 score as evaluation metrics.

### Model architecture

In the proposed model, the TCN module consists of three TCN layers with different scales, as depicted. Each TCN layer utilizes a different convolutional kernel size. The three channels of TCN employ kernel sizes of 3, 5, and 7, respectively. The preprocessed sensor data is fed into the multi-channel TCN layer, and a tensor (n, l, k) is defined. Here, ${\text{n}}$ represents the batch size, l represents the length of the selected sliding window, and k = 3 represents the $X$, $Y$, $Z$ axes of the acceleration, gyroscope, and magnetometer, respectively.

The input data is processed using the TCN module, which is a type of neural network designed for handling time series data. In comparison to the Convolutional Neural Network (CNN), TCN offers stronger temporal causality and a more flexible receptive field. The TCN module consists of three main components: causal convolution, dilated convolution, and residual convolution.

Causal convolution strictly adheres to the temporal order of the data. For instance, when considering data at time ${\text{t}}$, denoted as ${{\text{x}}}_{{\text{t}}}$, where ${\text{t}}={\text{n}}*{\text{l}}$, the prediction of ${{\text{y}}}_{{\text{t}}}$ depends solely on the data at time t and the preceding data. To illustrate this relationship, the data sequence ${\text{x}}_{0} ,{\text{x}}_{1} \ldots {\text{x}}_{{\text{t}}}$, xt is transformed to predict ${\text{y}}_{0} ,{\text{y}}_{1} , \ldots {\text{y}}_{{\text{t}}}$. The specific formula for this transformation is as follows:

1 $${\text{y}}_{0} ,{\text{y}}_{1} , \ldots {\text{y}}_{{\text{t}}} = {\text{f}}\left( {{\text{x}}_{0} ,{\text{x}}_{1} \ldots {\text{x}}_{{\text{t}}} } \right)$$

This issue often results in small receptive fields for causal convolutions. To address this, an expansion convolution is introduced to increase the receptive field. Dilated convolution, also referred to as dilated or atrous convolution, plays a vital role in this process. It incorporates an essential parameter known as the dilation factor, denoted as d. The formula for dilated convolution is as follows:

2 $${\text{F}}\left( {\text{t}} \right) = \mathop \sum \limits_{{{\text{i}} = 0}}^{{{\text{k}} - 1}} {\text{f}}\left( {\text{i}} \right) \cdot {\text{x}}_{{{\text{t}} - {\text{d}} \cdot {\text{i}}}}$$

**Figure 2:**
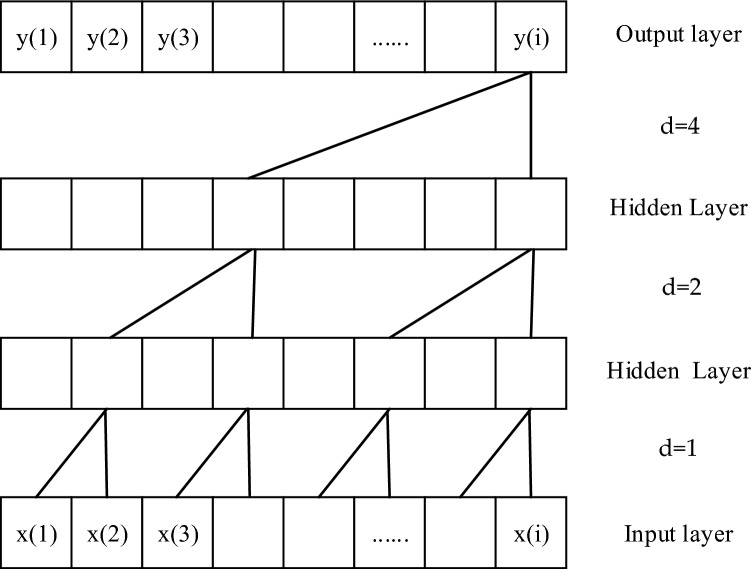
Convolutional diagram of expansion causality.

In the formula, ${\text{f}}({\text{i}})$ represents the ${\text{i}}$ th convolution coefficient, ${\text{k}}$ represents the size of the convolution kernel, and ${{\text{x}}}_{{\text{t}}-{\text{d}}\bullet {\text{i}}}$ represents the direction data before time ${\text{t}}$. When constructing the network, we set the expansion factor as d = bi, where i = 0,1,2,…n, usually the expansion factor is a multiple of 2. For example, as shown in Fig. 2, when the expansion factor is 2 and the number of network layers is 3, then d = 2i, i = 0, 1, 2.

**Figure 3:**
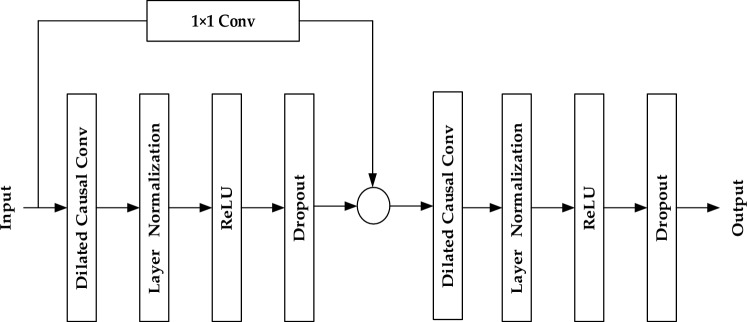
TCN residual module diagram.

The implementation of expansion convolution often necessitates additional network layers, which can lead to the problem of gradient vanishing. To address this issue, we introduce residual connections, Dropout, and Layer Normalization to construct a residual module within the TCN. The primary purpose of this module is to establish shortcut connections between network layers, effectively mitigating the problem of gradient vanishing associated with deep networks. The TCN residual module used in this paper is illustrated in Fig. 3. The formula for the residual connection is as follows:

3 $${\text{o}}={\text{Activation}}({\text{x}}+{\text{F}}\left({\text{x}}\right))$$

where ${\text{x}}$ is the input, ${\text{F}}({\text{x}})$ represents the residual map to be learned, and ${\text{o}}$ is the output of the layer.

The outputs from different channels, denoted as ${o}_{a}$, ${o}_{b}$, and ${o}_{c}$, with varying sizes, are concatenated. This concatenation process results in a combined TCN vector, represented as ht. The specific calculation formula for this operation is as follows:

4 $${h}_{t}=Concat({o}_{a},{o}_{b},{o}_{c})$$

**Figure 4:**
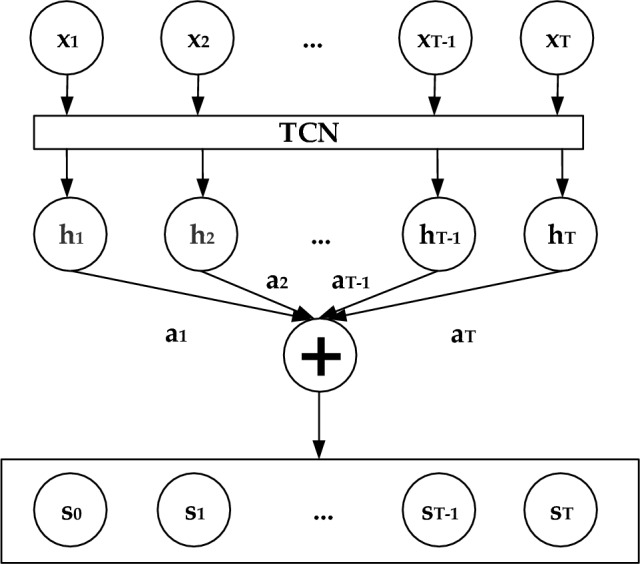
Structure of attention mechanism.

The attention mechanism, originally utilized in machine translation, has found wide application in various domains such as image processing, speech recognition, and natural language processing, thanks to the advancements in deep learning. In Fig. 4, ${{\text{x}}}_{{\text{t}}}$ (t ∈ [0, T]) represents the input sequence, ht (t ∈ [0, T]) represents the hidden layer input of the network, ${a}_{t}$ (t ∈ [0, T]) represents the attention weight values of the network, and ${s}_{t}$ (t ∈ [0, T]) represents the network output after incorporating attention. The specific formula for attention is as follows:

5 $${e}^{t}=Utanh(w{h}_{i}+b)$$

6 $${a}_{t}=\frac{exp({e}_{t})}{{\sum }_{j=0}^{t}{e}_{j}}$$

7 $${{\text{s}}}_{{\text{i}}}=\sum_{{\text{t}}}^{{\text{n}}}{{\text{a}}}_{{\text{i}}}{{\text{h}}}_{{\text{i}}}$$

where ${e}^{t}$ represents the attention weight calculated based on the network's output layer at time t. The attention weight is determined using weight parameters $U$ and $w$, along with a bias vector $b$. Ultimately, the classification of human activities is accomplished through the Softmax classification layer. The formula for this classification process is as follows:

8 $${\text{Softmax}}({{\text{z}}}_{{\text{i}}})=\frac{{e}^{{{\text{z}}}_{{\text{i}}}}}{{\sum }_{{\text{j}}=1}^{{\text{k}}}{{\text{e}}}^{{{\text{z}}}_{{\text{j}}}}}$$

where ${\text{z}}$ is the output of the softmax layer, and ${\text{k}}$ is the number of activity categories.

**Figure 5:**
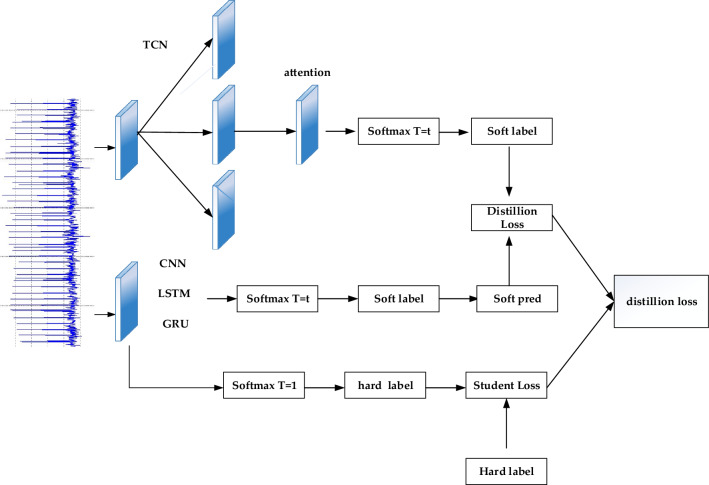
Knowledge distillation structure diagram.

As a model compression method, knowledge distillation, as shown in Fig. 5, mainly uses large and complex neural network models as teacher models, simple and lightweight neural network models as student models, and transfers the knowledge learned from the teacher model to the student model, significantly improving the accuracy of the student model. The student model can adjust distillation losses through temperature (T). Given the probability of ${\text{Softmax}}({z}_{i},{\text{T}})$, class $i$ is calculated based on Logit to obtain z_ I. The specific formula for adding the temperature softmax function is:

9 $${\text{Softmax}}({z}_{i},{\text{T}})=\frac{{\text{exp}}(z/T)}{\sum_{i}{\text{exp}}({z}_{i}/T)}$$

Therefore, the soft loss (${L}_{soft}$) makes Cross entropy for the softmax generated by the teacher model and the softmax generated by the student model, and hard (${L}_{hard}$) is the student loss of the standard softmax function. The complete Loss function L of knowledge distillation process is the weighted average value of soft loss and hard loss, which is defined as:

10 $$\begin{aligned} L_{soft} = & H\left( {softmax\left( {z_{t} ,T} \right),softmax\left( {z_{s} ,T} \right)} \right) \\ L_{hard} = & H\left( {softmax\left( {z_{s} ,1} \right),Y} \right) \\ L = & \alpha L_{soft} + \beta L_{hard} \\ \end{aligned}$$

where $H$ is the Cross entropy Loss function, ${z}_{t}$ and ${z}_{s}$ represents the logarithm of the teacher model and the student model,$\mathrm{\alpha }$ As the distillation loss coefficient, $\beta$ As a loss coefficient for students.

## Experiments

This section focuses on presenting the experimental setup and simulation results of the proposed model using the WISDM, PAMAP2, and USC-HAD datasets, which represent real-world scenarios. It is divided into four main parts: dataset introduction, data preprocessing, evaluation metrics, and results and discussion. The experiments were conducted in an environment based on a 64-bit Windows 11 operating system, equipped with an i7-11800H CPU running at 4.6 GHz and 64 GB of memory. The model experiments, training, and testing were performed using the TensorFlow 2.x framework.

### Dataset

To validate the effectiveness of the model, three datasets were utilized: WISDM^57^, Pamap2^58^, and USC-HAD^59^. Below is a description of the basic information for each dataset.

1. WISDM Dataset: This dataset is a publicly available dataset released by the Wireless Sensor Laboratory at Fordham University. It consists of 1,098,207 samples collected from 36 participants who wore Android smartphones in their front leg pockets. The triaxial acceleration data was recorded at a frequency of 20 Hz. The participants were instructed to perform six types of movements: sitting, standing, walking, going upstairs, going downstairs, and jogging.
2. Pamap2 Dataset: The Pamap2 dataset focuses on physical activity and human exercise data. It includes recordings of 18 exercises performed by 9 subjects, primarily ranging in age from 24 to 32 years old. The data collection phase involved the use of two accelerometers, a gyroscope, and a magnetometer, with a sampling rate of 100 Hz. The participants performed 12 activities, including lying down, sitting, standing, walking, running, cycling, Nordic walking, ironing, vacuuming, jumping rope, and going up and down stairs. Additionally, the participants were given six optional activities to choose from, which include watching TV, working on the computer, driving, folding clothes, cleaning the house, and playing football. For the experiments, 12 out of the 18 activities were used.
3. USC-HAD Dataset: The USC-HAD dataset utilizes a sensing platform called MotionNode to capture human signals. MotionNode is an inertial measurement unit (IMU) comprising a three-axis accelerometer and gyroscope, sampled at a frequency of 100 Hz. The IMU was worn by 14 participants, placed in a forearm bag on the right arm. The dataset encompasses a total of 12 activities, including walking forward, walking left, walking right, walking upstairs, walking downstairs, running forward, jumping, sitting, standing, sleeping, getting on an elevator, and getting off an elevator.

### Technical details

During the data processing stage, the original sensor data often contains noise and errors. To enhance the accuracy of training and prediction, a data cleaning technique is generally applied to eliminate incomplete and inaccurate data, including handling missing data. Subsequently, data normalization is performed to address the significant variation in sensor values.

The processed data is then segmented using a sliding window method. This segmentation approach plays a crucial role in dividing the data into the training and test sets. The selection of the sliding window size and the degree of overlap significantly impact the experiments' outcomes. For the WISDM, Pamap2, and USC-HAD datasets, the window size was set to 128, with a 50% overlap, taking into consideration the data frequency and human activity patterns. Specific optimal parameters: the size of convolution kernel is 64, the number of attention mechanism heads is 8, the learning rate is 0.0005, and the number of training epochs is 100, The ratio of the training set: test set is 8:2.

### Experimental evaluation index

Common indicators used in model classification include: Recall rate, accuracy, accuracy and F1 score will evaluate the performance of the model. Accuracy and accuracy are similar to the overall accuracy of judgments, but in the case of unbalanced samples, is not a good measure. The recall rate reflects the probability that the predicted correct sample accounts for the positive sample, and the F 1 score mainly plays the role of reconciling the accuracy rate and the recall rate. TP, TN, FP and FN are commonly used in model classification results. TP represents the number of correct samples with positive predictive value and TN represents the number of correct samples with negative predictive value and FN represents the number of wrong samples with positive predictive value and FN represents the number of wrong samples with positive predictive value. FP represents the number of error samples where the true value is negative and the predicted value is positive. For multi-classification work, FN is the true value is the error sample of the predicted value of this class is the error sample of the other class, and FP is the error sample of the other class is the error sample of the predicted value of this class.

The recall rate is the probability of being predicted to be a positive sample in an actual positive sample, expressed as follows:

11 $$Recall=\frac{TP}{TP+FN}$$

Accuracy is the ratio of the number of samples correctly classified by the classifier to the total number of samples in the original sample. Its expression is as follows:

12 $$Accuracy=\frac{TP+TN}{TP+FP+FN+TN}$$

Accuracy is for prediction and is the probability of actually being positive among all predicted positive samples, expressed as follows:

13 $$Precision=\frac{TP}{TP+FP}$$

The F1 score is a measure of the accuracy of the model on the dataset used to evaluate the binary classification, which is the average of accuracy and recall, expressed as follows:

14 $$F1=2\times \frac{Precision\times Recall}{Precision+Recall}$$

Confusion Matrix (CM) it is a square matrix that gives the full performance of the classification model. The rows of CM represent real class labels, and the columns represent predicted value labels.

### Hyperparameters are optimal

In order to obtain the optimal parameters of the model, this paper uses the number of convolution cores, the number of attention heads and the learning rate to adjust the model and select the most appropriate parameters.

**Figure 6:**
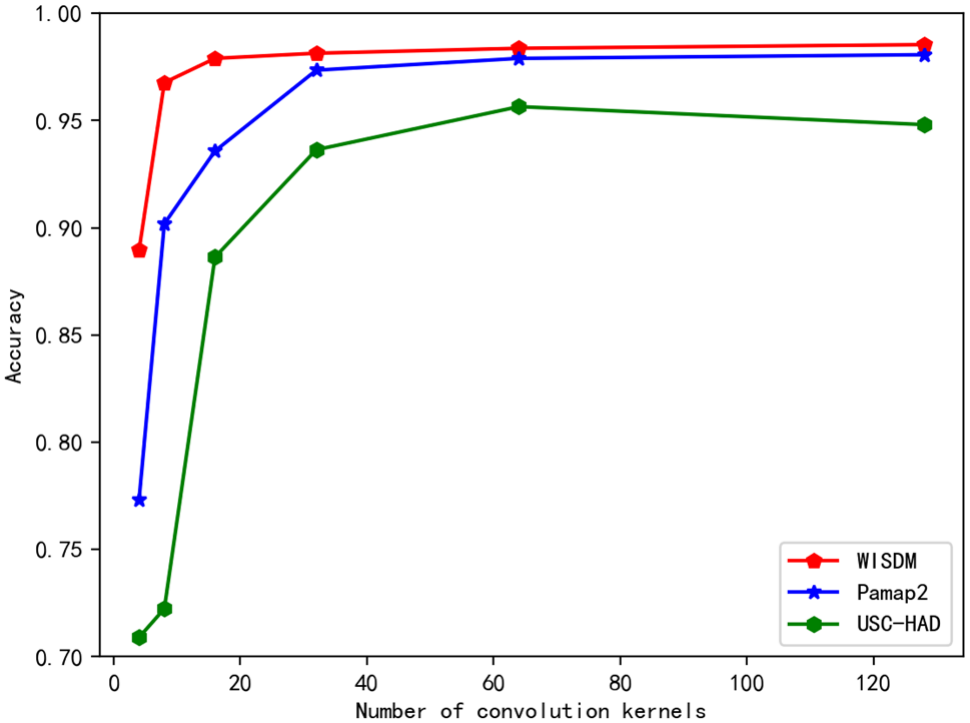
Knowledge distillation structure diagram.

First, the number of convolution nuclei is optimal. The size of convolution nuclei selected in this paper is 4, 8, 16, 32, 64, 128, and its accuracy is recorded. As shown in Fig. 6, it can be seen that when the convolution kernel is 32, the improvement is already very small, and the accuracy of 64 and 128 is basically unchanged. If the number of convolution is increased, the training cost will be increased. Therefore, in terms of the selection of the number of convolution kernel, 64 is chosen in this paper.

**Figure 7:**
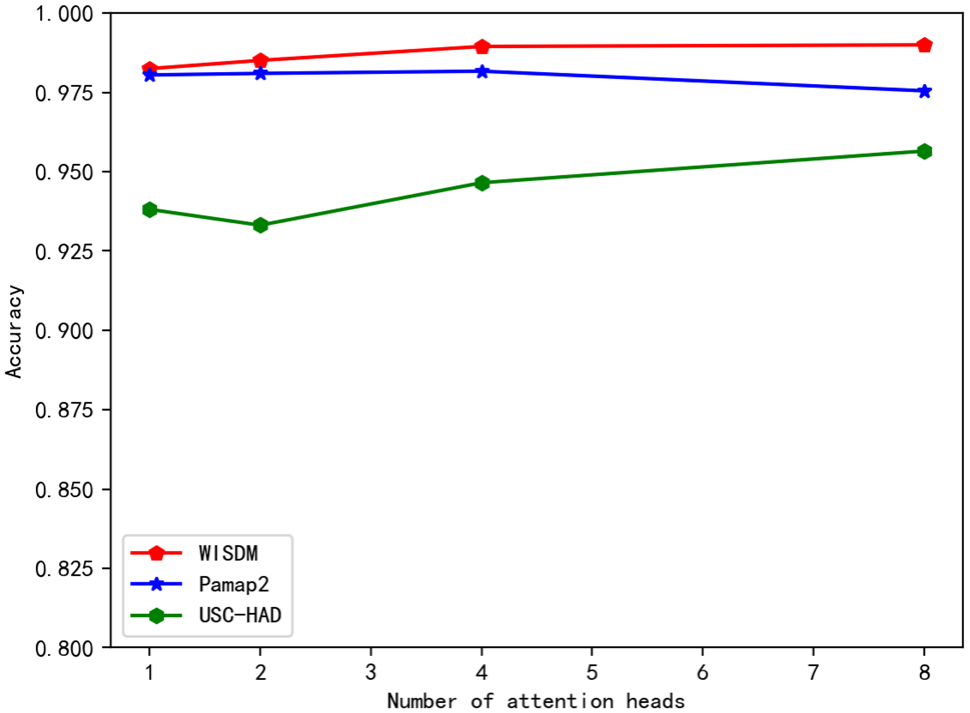
Influence of the number of attention heads on accuracy.

The number of attention heads selected in this paper is 1, 2, 4, 8, and its accuracy is recorded. As shown in Fig. 7, it can be seen that WISDM and USC-HAD data sets have a slight improvement from 4 to 8, while Pamap2 data sets have a downward trend. Therefore, in terms of the selection of the number of attention heads, 4 is chosen in this paper.

### Results and discussion

#### Comparison with state-of-the-art methods

Tables 1, 2 and 3 presents the evaluation metrics of the proposed model on the WISDM, PAMAP2 and USC-HAD datasets, respectively, including recall rate, accuracy, precision, and F1 score. From the observations, the TAHAR-Student-CNN model has the best performance on WISDM dataset, which outperformed its teacher model. Although the performance of the student model was similar to that of the teacher model on PAMAP2 and USC-HAD datasets, the performance of the student model also exceeded that of most models with less parameters. Overall, TAHAR-Teacher performs state-of-the-art in the three datasets, mainly due to strong TCN feature extraction and temporal correlation, surpassing GRU Attention and LSTM Attentions.

**Table 1:** Comparison of model performance across WISDM datasets.

Method	Recall	Accuracy	Precision	F1-score
SVM^60^	0.9049	0.9471	0.9258	0.9121
HMM^61^	0.9056	0.9436	0.9299	0.9128
Genetic algorithm^62^	0.9553	0.9724	0.9580	0.9565
GRU	0.9427	0.9676	0.9548	0.9484
GRU-attention^63^	0.9681	0.9790	0.9740	0.9710
CNN-GRU^64^	0.9377	0.9632	0.9457	0.9414
LSTM	0.9370	0.9600	0.9382	0.9371
Attention-LSTM	0.9398	0.9589	0.9442	0.9419
BiLSTM	0.9593	0.9734	0.9623	0.9607
CNN-LSTM^65,66^	0.9557	0.9711	0.9598	0.9576
CNN-BiLSTM^67^	0.9613	0.9740	0.9629	0.9620
CNN-A-BiLSTM^68^	0.9511	0.9656	0.9536	0.9521
CNN-BiGRU^69^	0.9648	0.9755	0.9645	0.9646
TAHAR-student-CNN	0.9880	0.9927	0.9865	0.9872
TAHAR-student-LSTM	0.9327	0.9604	0.9398	0.9342
TAHAR-student-GRU	0.9665	0.9804	0.9745	0.9701
TCN-attention-HAR-teacher	**0.9850**	**0.9903**	**0.9863**	**0.9856**

Significant values are in bold.

**Table 2:** Comparison of model performance across PAMAP2datasets.

Method	Recall	Accuracy	Precision	F1-score
SVM	0.8975	0.9162	0.9144	0.9046
HMM	0.9126	0.9266	0.9248	0.9173
Genetic algorithm	0.9271	0.9442	0.9427	0.9341
GRU	0.8948	0.9134	0.9138	0.9024
GRU-attention	0.9783	0.9792	0.9784	0.9783
CNN-GRU	0.9235	0.9372	0.9354	0.9288
LSTM	0.8749	0.8975	0.8965	0.8824
Attention-LSTM	0.8891	0.9116	0.9102	0.8972
BiLSTM	0.9120	0.9275	0.9259	0.9178
CNN-LSTM	0.9441	0.9527	0.9493	0.9464
CNN-BiLSTM	0.9363	0.9453	0.9462	0.9408
CNN-A-BiLSTM	0.9488	0.9555	0.9527	0.9507
CNN-BiGRU	0.9583	0.9652	0.9657	0.9616
TAHAR-student-CNN	0.9406	0.9496	0.9492	0.9442
TAHAR-student-LSTM	0.9584	0.9682	0.9689	0.9632
TAHAR-student-GRU	0.9446	0.9591	0.9620	0.9522
TCN-attention-HAR-teacher	**0.9837**	**0.9835**	**0.9823**	**0.9829**

Significant values are in bold.

**Table 3:** Comparison of model performance across USC-HAD datasets.

Method	Recall	Accuracy	Precision	F1-score
SVM	0.8200	0.8941	0.8641	0.8033
HMM	0.9015	0.9453	0.9199	0.9019
Genetic algorithm	0.9323	0.9556	0.9381	0.9345
GRU	0.7813	0.8461	0.8115	0.7905
GRU-attention	0.9420	0.9581	0.9431	0.9412
CNN-GRU	0.8394	0.8963	0.8485	0.8409
LSTM	0.7663	0.8428	0.7955	0.7709
Attention-LSTM	0.7945	0.8628	0.8252	0.7994
BiLSTM	0.8570	0.8946	0.8644	0.8577
CNN-LSTM	0.8988	0.9347	0.9030	0.8983
CNN-BiLSTM	0.9102	0.9448	0.9062	0.9005
CNN-A-BiLSTM	0.9176	0.9414	0.9110	0.9130
CNN-BiGRU	0.8884	0.9264	0.8860	0.8831
TAHAR-student-CNN	0.8987	0.8976	0.8482	0.8524
TAHAR-student-LSTM	0.8948	0.9317	0.9079	0.8836
TAHAR-student-GRU	0.8976	0.9317	0.8583	0.8701
TCN-attention-HAR-teacher	**0.9423**	**0.9632**	**0.9488**	**0.9434**

Significant values are in bold.

#### Impact of TCN mechanism

**Figure 8:**
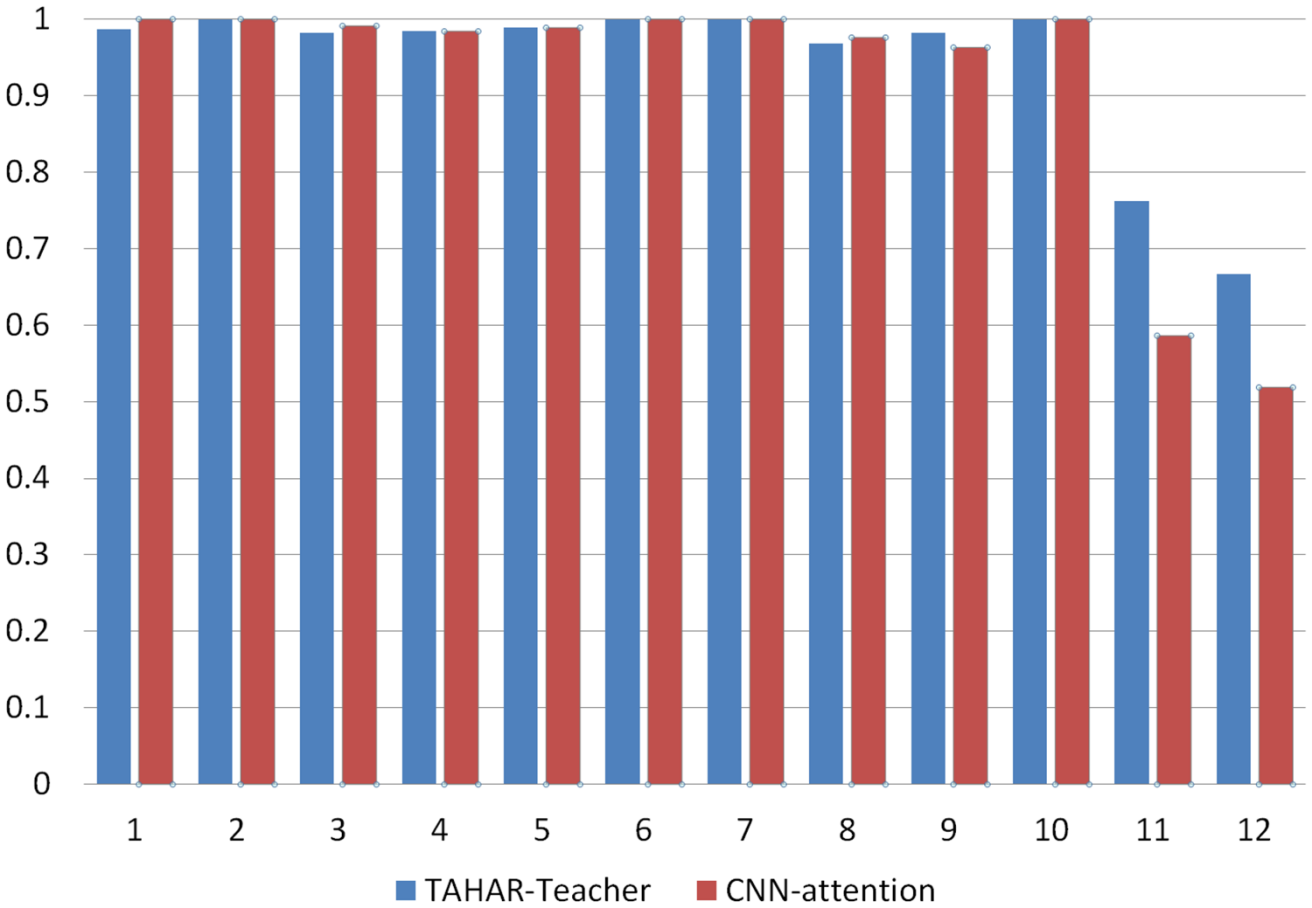
Comparison of F1 scores between the TACHAR-Teacher and CNN attention models on the USC-HAD dataset [i.e., Walking forward (1), walking left (2), walking right (3), going upstairs (4), walking downstairs (5), running forward (6), jumping (7), sitting (8), standing (9), sleeping (10), getting on the elevator (11), and getting off the elevator (12)].

As shown in Table 4, the multi-channel TCN attention model outperformed the multi-channel CNN attention model in all metrics. The improvement between these two models is particularly evident in the USC-HAD dataset. As illustrated in Fig. 8, this can be attributed to the opposite time patterns observed during elevator ascent and descent. Specifically, during the elevator descent process, the initial acceleration is downward, while the final acceleration is upward. On the contrary, during the elevator ascent process, the initial acceleration is upward, while the final acceleration is downward. The average sub window may lead to the loss of time information, leading to confusion between these two activities. However, by using TCN, the confusion between elevator ascent and descent can be significantly reduced.

**Table 4:** Comparison table of multi-channel TCN-attention-HAR and multi-channel CNN-attention on USC-HAD dataset.

Method	Recall	Accuracy	Precision	F1-score
CNN-attention	0.9111	0.9448	0.9136	0.9119
TCN-attention-HAR-teacher	**0.9423**	**0.9632**	**0.9488**	**0.9434**

Significant values are in bold.

#### Impact of attention mechanism

From Table 5, we can observe that the improvement in attention mechanism layer. It is mainly because the attention mechanism can assign weights for more important parameters, which verifies the effectiveness of attention mechanism.

**Table 5:** Comparison Table of the Recognition Effects of the Model with and without Attention Layers in this article.

Method	Recall	Accuracy	Precision	F1-score
TCN	0.9221	0.9556	0.9306	0.9070
TCN-attention-HAR-teacher	**0.9423**	**0.9632**	**0.9488**	**0.9434**

Significant values are in bold.

#### Impact of knowledge distillation

According to Table 6, three models with fewer parameters were selected, namely GRU, LSTM, and CNN models, as the student model. The proposed TAHAR model was used as the teacher model. The specific experimental results can be seen in Tables 1, 2 and 3. The distillation results of the three models (i.e., TAHAR-Student-CNN, TAHAR-Student-LSTM and TAHAR-Student-GRU) on the three datasets are better than other models in recognition performance, and are lower in parameters compared to other models. Among them, the CNN distillation results on the WISDM dataset also exceed the performance of the teacher model.

**Table 6:** Comparison table of various model parameters.

Method	Model parameters	Training time per epoch
GRU-attention	2,163,340	**18.797**
CNN-GRU	267,404	**6.974**
Attention-LSTM	2,180,240	18.832
BiLSTM	70.668	4.212
CNN-LSTM	348,810	**7.048**
CNN-BiLSTM	874,124	7.160
CNN-attention-BiLSTM	4,605,068	14.940
CNN-BiGRU	662,156	**7.717**
TAHAR-student-CNN	17,804	3066
TAHAR-student-LSTM	18,956	3.480
TAHAR-student-GRU	14,604	**3.472**
TAHAR-teacher	10,950,162	36.319

Significant values are in bold.

## Conclusions

This paper presents a deep learning model based on wearable sensing data for human activity recognition. By combining TCN and the Attention mechanism, a TCN-attention-HAR based model is constructed. Moreover, the knowledge distillation mechanism is utilized to reduce the model parameters with competitive performance. Experimental results among different models on three public datasets demonstrate that the proposed TRHAR exhibits favorable classification and recognition performance. It holds significant practical value in the field of human activity recognition and provides valuable insights for future research in this area.

## Data Availability

The WISDM datasets generated and/or analysed during the current study are available in the UCI Machine Learning Repository, https://archive.ics.uci.edu/dataset/507/wisdm+smartphone+and+smartwatch+activity+and+biometrics+dataset. The USC-HAD datasets generated and/or analysed during the current study are available in the USC Signal and Image Processing Institute, https://sipi.usc.edu/had/. The PAMAP2 datasets generated and/or analysed during the current study are available in the UCI Machine Learning Repository, https://archive.ics.uci.edu/dataset/231/pamap2+physical+activity+monitoring.
